# Integrated Computational Analysis of Genes Associated with Human Hereditary Insensitivity to Pain. A Drug Repurposing Perspective

**DOI:** 10.3389/fnmol.2017.00252

**Published:** 2017-08-08

**Authors:** Jörn Lötsch, Catharina Lippmann, Dario Kringel, Alfred Ultsch

**Affiliations:** ^1^Institute of Clinical Pharmacology, Goethe-University Frankfurt am Main, Germany; ^2^Fraunhofer Institute of Molecular Biology and Applied Ecology-Project Group, Translational Medicine and Pharmacology (IME-TMP) Frankfurt am Main, Germany; ^3^DataBionics Research Group, University of Marburg Marburg, Germany

**Keywords:** data science, computational biology, pain, humans, genetic variation, machine learning, perception, big data

## Abstract

Genes causally involved in human insensitivity to pain provide a unique molecular source of studying the pathophysiology of pain and the development of novel analgesic drugs. The increasing availability of “big data” enables novel research approaches to chronic pain while also requiring novel techniques for data mining and knowledge discovery. We used machine learning to combine the knowledge about *n* = 20 genes causally involved in human hereditary insensitivity to pain with the knowledge about the functions of thousands of genes. An integrated computational analysis proposed that among the functions of this set of genes, the processes related to nervous system development and to ceramide and sphingosine signaling pathways are particularly important. This is in line with earlier suggestions to use these pathways as therapeutic target in pain. Following identification of the biological processes characterizing hereditary insensitivity to pain, the biological processes were used for a similarity analysis with the functions of *n* = 4,834 database-queried drugs. Using emergent self-organizing maps, a cluster of *n* = 22 drugs was identified sharing important functional features with hereditary insensitivity to pain. Several members of this cluster had been implicated in pain in preclinical experiments. Thus, the present concept of machine-learned knowledge discovery for pain research provides biologically plausible results and seems to be suitable for drug discovery by identifying a narrow choice of repurposing candidates, demonstrating that contemporary machine-learned methods offer innovative approaches to knowledge discovery from available evidence.

## Introduction

Persistent pain is a major healthcare issue, as defined by WHO, affecting about a fifth of the European population increasing to a third in the over 70-year old (Elliott et al., 1999; Breivik et al., 2006). It has a highly complex pathophysiology (Julius and Basbaum, 2001; Schaible, 2007) and it is triggered by several different causes, such as cancer (Portenoy, 1992) and surgery (Kehlet et al., 2006). Therefore, the search for novel analgesic strategies is an active research topic receiving public funding (Kringel and Lötsch, 2015). In addition to the identification of novel drug targets from molecular research as the main line of research, scanning the existing pharmacopeia for repurposing candidates becomes increasingly successful (Ashburn and Thor, 2004). This is facilitated by developments in computational data science (President's Information Technology Advisory Committee, 2005).

An accepted source of novel options for the pharmacological treatment of (persistent) pain is the study of genes causally involved in hereditary syndromes with insensitivity to pain (Goldberg et al., 2012; Table 1). This set of genes has provided the targets of novel analgesics (Table 2). However, this unique set of genotypes also enables the study of key biological processes of pain from the perspective of the functions of the involved genes. As shown recently, similarities between the profiles of biological processes in which the genes coding for the targets of available drugs with the profile of the biological processes in which a given set of genes is involved can be employed for drug classification or repurposing (Lötsch and Ultsch, 2016a,b). Hence, picturing the genetic background of human insensitivity to pain could be explored for drug repurposing based on functional similarity in addition to the development of novel substances targeting some of the respective gene products (Table 2). The present analysis made extensive use of computational biology, knowledge discovery methods, publicly available databases and data mining tools (Table 3) to merge results from pain, genetics and pharmacological research.

**Table 1:** A set of *n* = 20 genes, in alphabetical order, that were reported to be causally associated with the hereditary human phenotype of insensitivity to pain.

**Gene**	**NCBI**	**Gene product**	**Syndrome**	**Syndrome code**	**OMIM**	**References**
*ATL1*	51062	Atlastin GTPase 1	Neuropathy, hereditary sensory, type ID	HSN1D	613708	Guelly et al., 2011
*ATL3*	25923	Atlastin GTPase 3	Neuropathy, hereditary sensory, type IF	HSN1F	609369	Kornak et al., 2014
*CLTCL1*	8218	Clathrin heavy chain like 1	Insensitivity to Pain with Preserved Temperature sensation		601273	Nahorski et al., 2015
*DNM1L*	10059	Dynamin 1 like	Encephalopathy, lethal, due to defective mitochondrial peroxisomal fission 1	EMPF1	614388	Sheffer et al., 2016
*EBF3*	253738	Early B-cell factor 3	Hypotonia, ataxia, and delayed development syndrome	HADDS	617330	Chao et al., 2017
*FAM134B*	54463	Family with sequence similarity 134, member B	Neuropathy, hereditary sensory and autonomic, type IIB	HSAN2B	613115	Kurth et al., 2009
*IKBKAP*	8518	Inhibitor of kappa light polypeptide gene enhancer in B-cells, kinase complex-associated protein	Dysautonomia, familial (Riley-Day syndrome)	HSAN III	223900	Anderson et al., 2001; Slaugenhaupt et al., 2001
*KIF1A*	547	Kinesin family member 1A	Hereditary sensory neuropathy type IIC	HSN2C	614213	Riviere et al., 2011
*LIFR*	3977	Leukemia inhibitory factor receptor alpha	Congenital pain insensitivity phenotype with progressive vertebral destruction			Elsaid et al., 2016
*MPV17*	4358	MpV17 mitochondrial inner membrane protein	Navajo neurohepatopathy	NNH	256810	Karadimas et al., 2006
*NGF*	4803	Nerve growth factor (beta polypeptide)	Neuropathy, hereditary sensory and autonomic, type V	HSAN V	608654	Einarsdottir et al., 2004
*NTRK1*	4914	Neurotrophic tyrosine kinase, receptor, type 1	Insensitivity to pain, congenital, with anhidrosis	HSAN IV	256800	Indo et al., 1996
*PRDM12*	59335	PR/SET domain 12	Insensitivity to Pain with hypohidrosis	HSAN VIII	616488	Chen et al., 2015
*RAB7A*	7879	RAB7A, member RAS oncogene family	Charcot-Marie-Tooth type 2B neuropathy	CMT2B	600882	Verhoeven et al., 2003; Janssens et al., 2014
*SCN11A*	11280	Nav1.9 (sodium voltage-gated channel alpha subunit 11)	Neuropathy, hereditary sensory and autonomic, type VII	HSAN VII	615548	Leipold et al., 2013; Woods et al., 2015
*SCN9A*	6335	Nav1.7 (sodium channel, voltage-gated, type IX, alpha subunit 9)	Insensitivity to pain, channelopathy-associated		243000	Cox et al., 2006
*SPTLC1*	10558	Serine palmitoyltransferase, long chain base subunit 1	Neuropathy, hereditary sensory and autonomic, type IA	HSAN1A	162400	Dawkins et al., 2001
*SPTLC2*	9517	Serine palmitoyltransferase, long chain base subunit 2	Neuropathy, hereditary sensory and autonomic, type IC	HSAN1C	613640	Rotthier et al., 2009
*TTR*	7276	Transthyretin	Carpal tunnel syndrome, familial	CTS1	115430	Swoboda et al., 1998
*WNK1*	65125	WNK lysine deficient protein kinase 1	Neuropathy, hereditary sensory and autonomic, type II (Morvan disease)	HSAN2A	201300	Lafreniere et al., 2004

*The genes were queried on March 13, 2017 from the “Online Mendelian Inheritance in Man” (OMIM) database at https://omim.org/ and the GeneCards database at http://www.genecards.org (Rebhan et al., 1997)*.

*HSAN, Hereditary sensory and autonomic neuropathy; HSN, Hereditary sensory neuropathy; OMIM, Online Mendelian Inheritance in Man database*.

**Table 2:** Novel analgesic drugs developed with the purpose to antagonistically target genes associated with human hereditary insensitivity to pain, i.e., to mimic the pain-insensitivity phenotype observed in carriers of loss-of-function mutations in these genes, being currently in a clinical phase of development according to publicly available sources of information (Table 3).

**Gene**	**Pharmacological target**	**Drug**	**Action**	**Company**
NGF	Nerve growth factor (beta polypeptide)	Tanezumab	Antibody	Pfizer
		MEDI-7352	Antibody	AstraZeneca
		Fasinumab	Antibody	Regeneron
		CRB-0089	Antagonist	Rottapharm Biotech
NTRK1	Neurotrophic tyrosine kinase, receptor, type 1	ASP-7962	Blocker	Astellas Pharma
		VM-902A	Blocker	Purdue Pharma
		ARRY-954	Blocker	Array BioPharma
		CRB-0089	Blocker	Rottapharm Biotech
		FX-007	Blocker	Flexion Therapeutics
SCN9A	Na_v_1.7 (sodium channel, voltage-gated, type IX, alpha subunit 9)	Funapide	Blocker	Xenon Pharmaceuticals
		CC-8464	Blocker	Chromocell
		DSP-2230	Blocker	Sumitomo Dainippon Pharma
		GDC-0310	Blocker	Genentech/Xenon Pharmaceuticals

*The information was queried on March 31, 2017 from the Thomson Reuters Integrity database at https://integrity.thomson-pharma.com*.

**Table 3:** Overview on data sources and computational tools used for the present data science approach to drug repurposing from knowledge about the functions of genes related to insensitivity to pain in humans.

	**Site name**	**URL**	**References**
Gene names and functions	AmiGO (search utility for GO)	http://amigo.geneontology.org/	Carbon et al., 2009
	Gene Ontology (GO)	http://www.geneontology.org/	Ashburner et al., 2000
	HUGO Gene Nomenclature Committee	http://www.genenames.org/	Seal et al., 2011
	NCBI gene index database	http://www.ncbi.nlm.nih.gov/gene/	
	GeneCards	http://www.genecards.org	Rebhan et al., 1997
Human diseases	Online Mendelian Inheritance in Man (OMIM®) database	http://www.ncbi.nlm.nih.gov/omim	
Drugs	DrugBank database	http://www.drugbank.ca	Wishart et al., 2006, 2008
	Thomson Reuters Integrity database	https://integrity.thomson-pharma.com	Nonfree
Software	R software	http://CRAN.R-project.org/	R Development Core Team, 2008

*All recourses are publically available, most of them free of charge*.

## Methods

**Figure 1:**
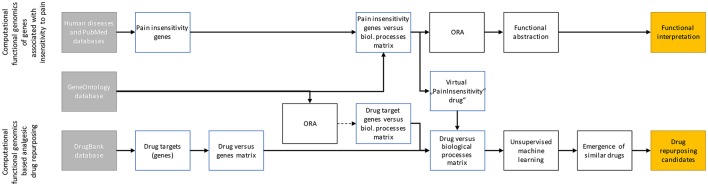
Scheme of the data analysis workflow. The analyses had two major aims, i.e., (i) assessing the biological functions of genes reportedly associated with insensitivity to pain (upper line) and (ii) using the biological processes in which these genes are involved to find repurposing candidates among DrugBank listed drugs (lower line). To this end, genes were identified in databases and their biological functions were associated based on the annotations in the Gene Ontology database; for the drug target coding genes (>4,000) with a filter for too many irrelevant terms implemented as an overrepresentation analysis. Such filter was not necessary for the only 20 genes associated with insensitivity to pain. However, for the latter, overrepresentation analysis (ORA) followed by functional abstraction was performed to obtain a comprehensible set of >10 biological functions which summarize the biological roles of these genes in an interpretable manner. The information obtained in the ORA of the 20 pain insensitivity genes was used to generate a virtual “pain drug” that was introduced into the “drug vs. biological processes” matrix of all drugs. Subsequently, unsupervised machine learning was used to find data structures among all drugs. Those drugs that, in the high dimensional vector space of associations with GO terms (biological processes), laid near the virtual “PainInsensitivity drug” were the repurposing candidates.

Data were analyzed using the R software package (version 3.3.3 for Linux; http://CRAN.R-project.org/; R Development Core Team, 2008). Following querying the relevant sets of genes by mining publicly available databases, the analyses aimed at (i) identifying the systems biology of hereditary insensitivity to pain as conferred by the functions of the causally involved genes and (ii) to explore whether this knowledge can be employed for drug repurposing approaches aimed at identifying potential candidates for the treatment of pain. The analytical steps are summarized in Figure 1 and described in full detail in the following paragraphs.

### Data mining

Genes involved in several different syndromes sharing the common phenotype of insensitivity to pain were queried on March 27, 2017 from the “Online Mendelian Inheritance in Man” (OMIM) database at (Online Mendelian Inheritance in Man, OMIM®, McKusick-Nathans Institute of Genetic Medicine, Johns Hopkins University, Baltimore, MD, USA) at https://omim.org/ and the GeneCards database at http://www.genecards.org (Rebhan et al., 1997) for “insensitivity to pain.” In addition, the medical literature was searched in the PubMed database at https://www.ncbi.nlm.nih.gov/pubmed. This provided a set of *n* = 20 genes (Table 1) that included genes causing human hereditary sensory and autonomic neuropathies (HSAN) and further genes for which published evidence is available for a causal implication with the common phenotype of insensitivity to pain.

To assess whether the known functions of the genes associated with human hereditary insensitivity to pain could be used for drug repurposing, genes coding for known molecular targets of known drugs were taken from the DrugBank database (version 5.0; http://www.drugbank.ca; Wishart et al., 2006, 2008). Specifically, a query of the DrugBank database on March 13, 2017, identified 4,834 drug entries, including 4,630 FDA-approved small molecule drugs, interacting with 2,215 unique targets. This provided the 4,834 × 2,215 “drug vs. gene” matrix.

### Assessment of the functions of genes associated with insensitivity to pain

The biological functions in which the products of genes associated with insensitivity to pain are involved were queried from the Gene Ontology knowledge base (GO; http://www.geneontology.org/; Ashburner et al., 2000). In this database, knowledge about the functions of genes is stored using a controlled and clearly defined vocabulary of GO terms (Camon et al., 2003) allocated to each gene (Camon et al., 2004). The GO database is searchable for three major categories, consisting of biological process, cellular component and molecular function. As the best representation of processes affected in hereditary insensitivity to pain as a potential source of therapeutic approaches, the GO category biological process was selected. According to the GO database, this category contains one or more ordered collections of molecular functions involving chemical or physical transformations such as cell growth and maintenance or signal transduction (Ashburner et al., 2000).

To capture biological processes that are particularly relevant to the present gene set, while eliminating data noise from common processes, the set of pain insensitivity genes was submitted to over-representation analysis (ORA) (Backes et al., 2007). This compared the occurrence (as defined by its annotation term) of the particular set of genes covered by a GO term with the number of genes expected to be defined by this term. The significance of the association of a GO term with the expected list of genes was determined by means of a Fisher's exact test (Fisher, 1922). The ORA attributed *p*-values to the resulting GO terms. A *p*-value threshold, *t*_*p*_, of 0.05 was applied and corrected for multiple testing using the false discovery rate (Benjamini and Hochberg, 1995).

The result was a representation of the complete knowledge about the biological roles of genes associated with insensitivity to pain in the form of a directed acyclic graph (DAG; Thulasiraman and Swamy, 1992). This depicted the biological processes as a higher level of organization of genes and signaling pathways described in a polyhierarchical structure where the processes are connected to each other by “is-a,” “part-of,” and “regulates” relationships (Ashburner et al., 2000). This result was used as the basis for a description of the functions of genes associated with insensitivity to pain. However, the complete DAG usually contained 136 GO terms that eluded intuitive interpretation (Miller, 1956). Therefore, the obtained results were transformed into a more intelligible form using the method of “functional abstraction” (Ultsch and Lötsch, 2014). This aims to reduce the numbers of GO terms using a heuristic search algorithm that identifies so-called “functional areas” (Ultsch and Lötsch, 2014), which are GO terms that qualify by their informational importance as headlines representing specific aspects (taxonomies) of the complete DAG with maximal coverage, precision, informational value and conciseness (Ultsch and Lötsch, 2014).

### Assessment of drug repurposing based on computational analysis

Following analysis of the biological processes in which the genes associated with human hereditary insensitivity to pain, the possibility was explored whether the discovered knowledge could be employed for a drug repurposing approach that uses functional similarity between drugs and key functions of a trait-relevant gene set (Lötsch and Ultsch, 2016a,b). This was based on (i) the biological processes identified as characterizing the genes associated with insensitivity to pain obtained in above analysis, and required in addition (ii) associating drugs, via the genes coding for their targets, with biological processes followed by (iii) the identification of those drugs that are associated with similar biological processes that characterize the genes associated with insensitivity to pain phenotypes. These analytical steps will be described in as follows.

As described previously (Lötsch and Ultsch, 2016a,b), the association of drugs with biological processes was obtained by combining drugs which are associated with molecular targets available from the DrugBank database with genes associated with biological processes as queried from the Gene Ontology database (Table 3). Specifically, a set of *n* = 2,215 genes queried as drug targets from the DrugBank database was submitted to overrepresentation analysis to identify relevant biological processes, expressed as GO terms, which can be addressed by the available drugs. ORA, performed as described above, however, with the parameters *t*_*p*_ = 1·10^−15^ and α correction according to Bonferroni (1936), provided 830 GO terms that can be considered as specifically describing the biological processes in which 2,215 targets of the drugs known to the DrugBank database are involved. This resulted in a 2,215 × 830 “gene vs. biological process” matrix. Here, in contrast to its use for functional interpretation of a gene set applied onto the pain insensitivity genes as described above, the ORA served merely as a filter for relevant GO terms, which was set at a conservative *p*-value threshold to eliminate too many generic terms from the matrix; as a functional interpretation of the biological roles of all drug targets was not intended, functional abstraction was not applied. From the matrix product of the “drug vs. genes” matrix and the “genes vs. processes” matrix, a 4,834 × 830 “drug vs. biological process” matrix was provided.

Subsequently, a virtual drug “PainInsensitivity” was added to the “drug vs. biological process” matrix. This “drug” carried a single vector composed of the numbers that indicate how often particular biological processes have been associated with a member of the *n* = 20 sized gene set causally involved in hereditary insensitivity to pain. Using the biological processes that were addressed by both, DrugBank queried drugs and the “PainInsensitivity” drug, a 4,512 × 38 sized “drug vs. biological process” matrix was obtained. Within this matrix, the Euclidian distances of each of the 4,511 DrugBank annotated drugs to the “PainInsensitivity” drug were calculated.

To eliminate drugs dissimilar to the “PainInsensitivity” drug, the reciprocals of the Euclidian distances were submitted to calculated ABC analysis (Ultsch and Lötsch, 2015). This is a categorization technique for the selection of a most important subset among a larger set of items. It divides a set of positive data into three disjoint subsets “A,” “B,” and “C.” Subset “A” comprises the profitable values, i.e., “the important few” (Pareto, 1909; Juran, 1975) and is found using the x-value where the slope of an ABC curve (Gastwirth and Glauberman, 1976), i.e., a plot of cumulative distribution of items sorted in decreasing order of magnitude, takes a value of 1. These calculations were done using our R package “ABCanalysis” (http://CRAN.R-project.org/web/packages/ABCanalysis/index.html; Ultsch and Lötsch, 2015). To exclude drugs at large distances from the “PainInsensitivity” drug, ABC analysis was performed in a nested manner, i.e., ABC set “A” of a first analysis was re-submitted to a second ABC analysis. This provided a 414 × 38 sized “drug vs. biological process” matrix.

In this 414 × 38 sized “drug vs. biological process” matrix, data structures were analyzed to identify a cluster of DrugBank annotated compounds located in the vicinity to the “PainInsensitivity” drug. This was obtained using unsupervised machine learning (Murphy, 2012). Specifically, each drug is represented as a vector in a *d* = 38-dimensional feature space of positive associations with biological processes. A projection and visualization method was used that projects the d-dimensional feature space onto a two-dimensional plane and depicts the structures of the feature space in form of a landscape. A self-organizing artificial neuronal network of Kohonen (1982) type emergent SOM, (ESOM) (Ultsch and Sieman, 1990; Lötsch and Ultsch, 2014) was used. The neural network consisted of a two-dimensional toroid grid (Ultsch, 2003) of 50 × 80 neurons (*n* = 4,000 units). Each neuron holds, in addition to the input vector from the high-dimensional space of processes associated to each drug, a further vector carrying “weights,” which were initially randomly drawn from the range of the data variables and subsequently adapted to the data during the learning phase that used 50 epochs.

The trained emergent self-organizing map (ESOM) represents the drugs on a two-dimensional toroid map as the localizations of the “best matching units” (BMU). On top of this grid the distance structures in the high-dimensional feature space of biological processes is depicted in form of a so-called U-Matrix (Ultsch and Sieman, 1990; Lötsch and Ultsch, 2014). The machine learning was performed using the R library “UMatrix” (M. Thrun et al., Marburg, Germany, publicly available at http://www.uni-marburg.de/fb12/datenbionik/softwareweb/packages/ABCanalysis/index.html. Only the cluster was further regarded that included the “PainInsensitivity” drug. For the DrugBank annotated members of this cluster, evidence was queried from the literature supporting, or discouraging, a possible involvement in pain.

## Results

### Computational analysis of the genes involved in hereditary insensitivity to pain

To identify the systems biology of hereditary insensitivity to pain, a set of *n* = 20 unique genes (Table 1) for which published evidence supported an association with the human phenotype of inherited insensitivity to pain was queried form publicly available databases (Table 3). The biological functions associated with the expression of these genes and their respective products were queried from the Gene Ontology knowledge base (GO; http://www.geneontology.org/; Ashburner et al., 2000). Over-representation analysis (ORA) against all human genes (*n* = 18,750 in the used GO version) and using an FDR corrected *p*-value threshold of *t*_*p*_ = 0.05 resulted in 136 significant GO terms (Supplementary Figure 1).

Subsequent functional abstraction, which is a method developed to reduce a large set of GO terms to a comprehensible small subset of “headline terms” or “functional areas” that represent specific aspects (taxonomies) of the complete polyhierarchy with maximal coverage, precision, informational value and conciseness (Ultsch and Lötsch, 2014), identified 8 GO terms qualifying as headlines to summarize the biological functions that are particularly important addressed by the 20 genes associated with insensitivity to pain, among all human genes (Table 4). A GO annotation with pain was found in two particular taxonomies of the polyhierarchy, i.e., under the headline “multicellular organismal response to stress” (GO:0033555) that exclusively included response to pain (GO:0048265) and its descendants, and again under the headline “Neurological system process” (GO:0050877), which at the chosen *p*-value threshold ended downstream with “sensory perception of pain” (GO:0019233) and “neuronal action potential” (GO:0019228).

**Table 4:** Functional areas representing the genetic background of hereditary insensitivity to pain presented in a polyhierarchy of GO terms with a maximum of certainty, information value, coverage and conciseness (Ultsch and Lötsch, 2014).

**GO term ID**	**Functional area (GO term of category biological process)**	**No. of genes**
GO:0033555	Multicellular organismal response to stress	2
GO:0070997	Neuron death	3
GO:0050877	Neurological system process	5
GO:0009991	Response to extracellular stimulus	5
GO:0007399	Nervous system development	6
GO:0071704	Organic substance metabolic process	11
GO:0016043	Cellular component organization	13
GO:0065007	Biological regulation	17

*Specifically, significant gene ontology (GO) terms were obtained by means of over-representation analysis (ORA) of the 20 genes against all human genes. The precise definition of the GO terms can be obtained using the AmiGO search tool for GO at http://amigo.geneontology.org/ (Carbon et al., 2009)*.

A second larger group of biological processes typically annotated with the genes associated with insensitivity to pain comprised developmental and structural aspects of the nervous system, i.e., “nervous system development” (GO:0007399) and “neuron death” (GO:0070997) including “negative regulation of apoptotic process” (GO:0043524) as a downstream terminal of the taxonomy, and also processes of “cellular component organization” (GO:0016043). A third larger group of biological processes typically annotated with the genes associated with insensitivity to pain comprised metabolic processes (“organic substance metabolic process,” GO:0071704), which included biosynthetic processes related to sphingolipids (“sphingosine biosynthetic process,” GO:0046512) and ceramides (“ceramide biosynthetic process,” GO:0046513), “nerve growth factor processing” (GO:0032455), “positive regulation of lipophagy” (GO:1904504), and “positive regulation of histone H3-K9 dimethylation” (GO:1900111). The remaining processes could only be summarized in large sets of more heterogeneous biological functions such as “biological regulation.”

### Candidate drugs for repurposing

To explore whether the results of the computational analysis of genes associated with human insensitivity to pain could be employed for drug repurposing, a virtual drug “PainInsensitivity” was created that carried a single vector composed of the numbers of how often particular biological processes have been associated with a member of the *n* = 20 genes causally involved in hereditary insensitivity to pain. Only the *n* = 38 biological processes were included that were also associated with any drug queried from the DrugBank database. Similarly, vectors coding for the associations with biological processes were assigned to each drug. The data space was reduced by eliminating drugs at large Euclidian distances from the “PainInsensitivity” drug, using nested ABC analysis.

**Figure 2:**
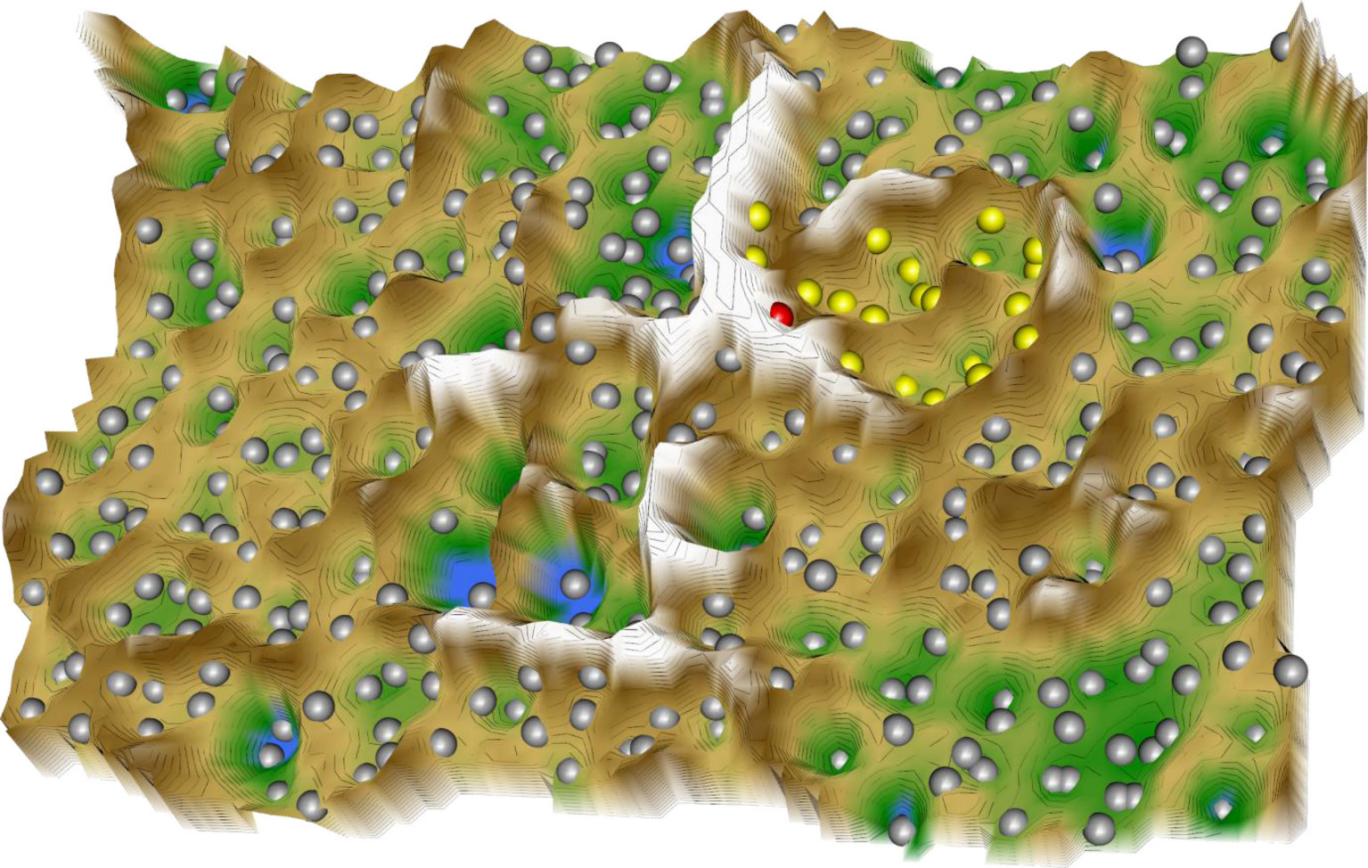
3D-view of the U-matrix visualization of distance based structures of the 414 × 38 sized “drug vs. biological process” matrix, comprising the 413 drugs annotated with one or more of the *n* = 38 biological processes assigned to both, the set of 20 genes causally implicated in insensitivity to pain and the drug targets queried form the DrugBank database, which following ABC analysis based item selection were found in at closer Euclidian distances form the virtual “PainInsensitivity” drug (red dot) that carried all of the *n* = 38 processes. The U-matrix has been obtained using a projection of the data points onto a toroid grid of 4,000 neurons where opposite edges are connected. The U-Matrix was colored as a geographical map with brown (up to snow-covered) heights and green valleys with blue lakes. Valleys indicate clusters and watersheds indicate borderlines between different clusters. The dots indicate the so-called “best matching units” (BMUs) of the self-organizing map (SOM), which are those neurons whose weight vector is most similar to the input. A single neuron can be the BMU for more than one data point; hence, the number of BMUs may not be equal to the number of drugs. In the vicinity of the red dot, i.e., the virtual “PainInsensitivity” drug, a mount ridge surrounded valley was observed that represented a cluster of drugs (yellow dots) most similar to the virtual “PainInsensitivity” drug. However, the latter was located eccentrically in the cluster indicating that the similarity to any of the DrugBank queried repurposing candidates for pain therapy is incomplete. The other drugs (gray dots) lay outside the cluster of the “PainInsensitivity” drug and could therefore be rejected as repurposing candidates based on the present approach to search for drugs that with respect to the addressed biological processes are most similar to the pattern of biological processes in which the genes associated with insensitivity to pain are involved. The figure has been created using the R software package (version 3.3.3 for Linux; http://CRAN.R-project.org/; R Development Core Team, 2008) using our R library “Umatrix” (M. Thrun, F. Lerch, Marburg, Germany, http://www.uni-marburg.de/fb12/datenbionik/software; file http://www.uni-marburg.de/fb12/datenbionik/umatrix.tar.gz).

Following projection of the resulting 414 × 38 sized “drug vs. biological process” data space onto a toroid grid of 50 × 80 neurons and training of a self-organizing map, a U-matrix visualization was displayed on top of this SOM (Figure 2). Large U-heights in this visualization indicated a large gap in the data space whereas low U-heights indicated that the points are close to each other in the data space. The distance-based data structure indicated a cluster that comprised the virtual drug “PainInsensitivity” (red dot in Figure 2) together with further *n* = 22 DrugBank listed substances (Table 5). These substances can be considered as repurposing candidates for pain, based on their vicinity (yellow dots in Figure 2) in the high-dimensional data space to the biological processes associated with the genes causally involved in insensitivity to pain as represented on the SOM by the “PainInsensitivity” drug.

**Table 5:** Candidate DrugBank listed substances that qualify for repurposing as treatments of pain, according to the similarity between the biological processes associated with the *n* = 20 genes causally involved in human insensitivity to pain and captured in the virtual “PainInsensitivity” drug, and the biological processes in which the 413 drugs queried from the DrugBank database (Wishart et al., 2006, 2008) are involved.

**Drug name**	**DrugBank ID**	**Evidence supporting involvement in pain**
“PainInsensitivity” drug	none	Not applicable (virtual drug)
Acetylsalicylic acid	945	Approved analgesic
Myristic acid	8231	Antinociceptive effects (Intahphuak et al., 2010)
Phosphonoserine	4522	–
Tamoxifen	675	–
Adenosine monophosphate	131	Its inhibition attenuated pain (Liou et al., 2007)
Phosphonothreonine	2482	–
Imatinib	619	Restored morphine analgesic potency (Donica et al., 2014)
Sorafenib	398	Might induce pain (Di Cesare Mannelli et al., 2015)
Flavopiridol	3496	Recovery from tactile allodynia (Tsuda et al., 2011)
Nintedanib	9079	–
Ellagic acid	8846	Antinociceptive effects (Mansouri et al., 2014)
Dasatinib	1254	–
Sunitinib	1268	Hyperalgesic effects (Bullon et al., 2016)
Staurosporine	2010	Counteracting capsaicin sensitization (Anand et al., 2015)
Lenvatinib	9078	–
Pazopanib	6589	–
MP470	5216	–
ABT-869	6080	–
XL999	5014	–
Yohimbine	1392	Antinociceptive effects (Shannon and Lutz, 2000)
Ponatinib	8901	–
Caffeine	201	Analgesic effects shown and discussed (Baratloo et al., 2016)

*The 22 drugs are the members of the cluster in the high dimensional space to which the virtual “PainInsensitivity” drug belonged (Figure 2)*.

## Discussion

The present analysis used empirical evidence for human genes that when nonfunctional may cause the phenotype of insensitivity to pain. The set of *n* = 20 genes included those causally involved in hereditary sensory and autonomic neuropathies and for long in the center of pain research, and in addition further genes involved in several different neurological syndromes or, such as *SCN9A*, genes that when nonfunctional are mainly associated with insensitivity to pain. Indeed, the only additional phenotype in subjects carrying loss-of-function mutations in *SCN9A* was anosmia (Cox et al., 2010). In the present analysis, the common phenotype of insensitivity to pain associated with the complete gene set was used to address the functional background of pain insensitivity and to explore a computational attempt at using the information for drug repurposing. The computational approach allowed using data on the biological functions of genes acquired in any context, without restriction to pain research (Lötsch et al., 2013).

The computational analysis of genes associated with human insensitivity to pain included, in addition to basic functions as neuronal signaling or the perception of pain, as major components (i) nervous system development and structural component assembly and (ii) lipid-mediator based signaling including sphingosines and ceramides. Nervous system development, while presently probably also associated with the hereditary developmental disorders of the nervous system, appear to be a consistent part of the genetic background of pain since they also emerged as particularly prominent systemic functions of larger sets of genes related to all aspects of pain (Ultsch et al., 2016). As discussed there, as possible explanation of the consistent appearance of nervous system development among key biological processes exerted by pain-relevant sets of genes involves the concept of chronic pain as a dysregulation in biological processes that describe its systemic features of learning and neuronal plasticity (Mansour et al., 2014). Thus, the structural aspect of nervous system development would be compatible with both, hereditary developmental neuropathies and pain.

A role of lipid signaling is compatible with prior knowledge of the pathophysiology of pain. Indeed, the ceramide-to-sphingosine pathway has been proposed already as a therapeutic target in pain (Salvemini et al., 2013). Similarly, sphingosine-1-phosphate induced nociceptor excitation and ongoing pain behavior in mice and humans (Camprubi-Robles et al., 2013) and therefore, sphingosine-1-phosphate receptors have been proposed as novel targets for the treatment of pain (Welch et al., 2012). While these findings have emerged from basic research, the present prominent role of this pathway highlights the suitability of the computational approach analyzing available information about a particular subset of pain-related genes. Again, the biological plausibility applies to both, developmental neuropathies and pain.

The idea behind using the knowledge about the biological processes characterizing insensitivity to pain for drug repurposing is to use the similarity measure in the high-dimensional vector space of the drug's interactions with biological processes for the identification of substances qualifying for the treatment of traits defined on the basis of biological processes (Lötsch and Ultsch, 2016b). A disease-relevant gene set is functionally analyzed and compared with the biological processes in which the targets of available drugs are involved. This approach presently resulted in the selection of a subset of *n* = 22 substances, which is the size of the cluster that surrounded the functional information of the present trait of interest in the high-dimensional data space (Figure 2). Results obtained with the present computational approach require basic research verification. However, the set included substances for which such evidence could be found. For example, myristic acid is contained at 18.64% in coconut oil, which when orally administered to rats produced moderate anti-inflammatory and anti-nociceptive effects (Intahphuak et al., 2010). Imatinib, a tyrosine kinase inhibitor, had no antinociceptive effects in a nerve injury model in rats when administered alone; however, when combined with a previously ineffective dose of morphine, complete pain relief was obtained (Donica et al., 2014), which was attributed to its platelet-derived growth factor inhibiting actions (Mcgary et al., 2004).

Similarly, flavopiridol or alvocidib, an inhibitor of cyclin-dependent kinases, was shown to facilitate the recovery from tactile allodynia when in a rat nerve injury model, which was attributed to its Janus kinase pathway inhibiting actions (Tsuda et al., 2011). Ellagic acid, a polyphenolic compound from plants such as raspberries, eucalyptus, and nuts (Clifford and Scalbert, 2000), showed antinociceptive effects and potentiated the effects of morphine in different rat models of pain (Mansouri et al., 2014). Furthermore, staurosporine, an alkaloid initially isolated from the bacterium *Streptomyces staurosporeus* (Omura et al., 1977) and found to inhibit protein kinases, inhibited Angiotensin II mediated sensitization of post mortem analyzed human nerves (Anand et al., 2015), which is in line with the targeting of angiotensin 2 type II receptors as a novel treatment for neuropathic pain (Rice et al., 2014).

However, the list of candidate drugs for repurposing also included the multitargeted receptor tyrosine kinase inhibitors sorafenib and sunitinib, for which evidence suggests hyperalgesic actions. This emphasizes that, as discussed previously (Lötsch and Ultsch, 2016b), a limitation of the present implementation of this computational approach to drug repurposing consist of the unsigned inclusion of drug vs. target interactions, i.e., without distinction of agonistic from antagonistic actions. Therefore, topical experts' knowledge is required to amend this limitation. Of further note, the present computational approach at drug repurposing based on computational analysis of gene functions and similarity measures in the high-dimensional data space still depends on the accuracy and completeness of the information in the queried databases. This makes it vulnerable to research bias and erroneous information in the databases, however, the increasing trend toward “big data” supports the expectation of a continuously broadening knowledge base.

The present computational analysis of the genes involved in human hereditary insensitivity to pain in a drug repurposing context extends previous applications of the recently proposed concept of “process pharmacology” (Lötsch and Ultsch, 2016b) by (i) the inclusion of a topically selected set of genes in addition to a previously used set of genes derived from microarray analysis, and (ii) the introduction of the concept of a “virtual drug,” i.e., a vector of biological processes representing the key functions of a gene set that can be entered as a further drug into the drug vs. biological process matrix, thereby facilitating the search for repurposing candidates by using similarity measures in the high dimensional vector space. As described previously (Lötsch and Ultsch, 2016a,b), in the present approach diseases are regarded as resulting from the activity of pathophysiological processes captured in the GO database via the category “biological processes,” i.e., series of events or molecular functions with a defined beginning and end (Ashburner et al., 2000). Such a focus on disease relevant biological processes (Lötsch and Ultsch, 2016b) has been shown to provide a robust basis for drug classification agreeing with classical approaches (Lötsch and Ultsch, 2016a) and may provide a phenotypic approach to drug discovery and repurposing.

The present ORAs were performed with different *p*-value thresholds. The conservative *p*-value threshold of *t*_*p*_ = 1·10^−15^ used for the analysis of the drug targets was heuristically chosen to accommodate the intention to only include highly relevant terms and to obtain a set size of 500–1,000 terms that had proven suitable in previous similar analyses (Lötsch and Ultsch, 2016a,b). By contrast, the *p*-value threshold of 0.05 chosen for the ORA of the *n* = 20 genes associated with human insensitivity to pain was chosen for practical reasons. That is, it was the strictest criterion providing the generally accepted significance level of *p* = 0.05 and a correction for multiple testing, a stricter criterion or the use of more conservative α-correction according to Bonferroni (1936) led to a nearly empty ORA result for the present set of *n* = 20 genes involved inhuman insensitivity to pain and could therefore not applied. However, a systematic test of the optimum *p*-value threshold for the present computational drug repurposing approach remains an active research topic to be addressed in future work.

By placing the modulation of biological processes into the focus with molecular targets merely serving as intermediates, the present concept may exceed the particular molecular mechanism by which a drug acts making it particularly suitable for drug repurposing by eliminating the restriction to a specific molecular target. In this respect, the present concept complements non-redundantly alternative implementations of computational science in drug research and development. For example, the analysis of molecular interaction networks (e.g., protein-protein interaction networks or gene-gene co-expression networks) may be employed to study the possible consequences of target removals, considering a target as effective when its removal modifies the network in an essential way (Penrod et al., 2011). Other approaches proposed to use supervised machine learning, i.e., support vector machines, to analyze druggable protein-protein interaction on the basis of the number of shared GO terms indicating similarities in biological function between two interacting proteins (Sugaya et al., 2007; Sugaya and Ikeda, 2009). Further approaches include pathway based analysis where pathway annotations, such as provided by the Kyoto Encyclopedia of Genes and Genomes (KEGG) pathway database are “translated into a two-dimensional statistical test problem that involves Wilcoxon's signed rank sum test in order to compute a Z-score for each pathway that quantifies the degree of alteration across the different experimental conditions” (Herwig and Lehrach, 2006). These approaches have also shown to provide clinically useful approaches to drug development such as the identification of anticancer drug combinations (Azmi et al., 2010), which complements a similar success of the present method (Lötsch and Ultsch, 2016b). This emphasizes the increasing utility of a variety of computational approaches to gene functions in drug research, for which the present analysis adds further support.

Most contemporary machine learning techniques for the analysis of genomic data are of the supervised learning type (for a recent review, see Libbrecht and Noble, 2015). These methods aim at the diagnosis (classification) of cases. By contrast, in the present work unsupervised methods were used, which mainly aim at structure detection in high dimensional (genetic) data. Typical methods for structure detection in high dimensional data are (i) nonlinear projections such as multidimensional scaling (MDS) (Tzeng et al., 2008), t-SNE (van der Maaten and Hinton, 2008; Bushati et al., 2011) or curvilinear component analysis (Alanis-Lobato et al., 2015) and (ii) clustering methods summarized in (Pirim et al., 2012). The ESOM/Umatrix method used here can be regarded as a combination of a disentangling and neighborhood preserving projection method combined with a clustering algorithm. This method has been demonstrated to be superior to other approaches in the sense, that complex and intertwined clusters can be identified, however, no spurious structures are artificially introduced by the clustering method itself (Ultsch and Lötsch, 2017) for which a superiority to many other projection methods has been shown (Tasdemir and Merényi, 2012).

## Conclusions

Genes causally involved in human insensitivity to pain provide a unique source of studying the pathophysiology of pain and the development of novel analgesic drugs. In keeping with the contemporary trend toward “big data” analysis in biomedical research, an integrated computational analysis was performed to study the set of genes for emergent, principal pathophysiological processes that characterize insensitivity to pain. As a result, a particular importance for pain perception was observed for processes related to structural changes in the nervous system and to ceramide and sphingosine signaling pathways, which is compatible with suggestions of using these pathways as therapeutic target in pain. Using the biological processes characterizing hereditary insensitivity to pain for drug repurposing, a clear cluster of *n* = 22 substances emerged that comprised several drugs for which implications in pain have been shown occasionally in preclinical experiments. Thus, the present concept provides biologically plausible results and seems to be suitable for drug discovery by identifying a narrow choice of repurposing candidates, demonstrating that contemporary machine-learned methods offer innovative approaches to knowledge discovery from previous evidence.

## Author contributions

Wrote the paper, conception or design of the work: JL. Analyzed the data: JL and CL. Data collection: DK and JL. Provided advice for data analysis: AU.

### Conflict of interest statement

The authors declare that the research was conducted in the absence of any commercial or financial relationships that could be construed as a potential conflict of interest.

## Supplementary material

Supplementary Figure 1 This figure shows the top-down representation of the GO terms representing the biological processes in which the set of *n* = 20 genes causally involved in hereditary absence of pain perception (Table 1) is involved (for an enlarged version, see the Supplementary Figure). The graphical representation follows the standard of the GO knowledgebase, where GO terms are related to each other by “is-a,” “part-of,” and “regulates” relationships forming a polyhierarchy organized in a directed acyclic graph (DAG, Thulasiraman and Swamy, 1992). The figure represents the results of an over-representation analysis with parameters for the *p*-value threshold, *t*_*p*_ = 0.05 and FDR α-correction. **(Top)** Significant terms are shown as colored circles with the number of member genes, the number of expected genes by chance and the significance of the deviation in the observed from the expected number of genes indicated (yellow = headline, red = significant term located in the polyhierarchy below a functional area). Blue vertices or blue labels, are the most specific terms (leaves of the DAG) at the end of a taxonomy in the polyhierarchy. The biological processes in which these genes are involved can be summarized by seven primary “functional areas” representing the most remarkable nodes with respect to their localization in the polyhierarchy. **(Bottom)** Zoomed parts of particular functional areas, recreated in a slightly different arrangement to enhance visibility.

## References

[B1] Alanis-Lobato G. Cannistraci C. V. Eriksson A. Manica A. Ravasi T. (2015). Highlighting nonlinear patterns in population genetics datasets. Sci. Rep. 5:8140. 10.1038/srep08140 25633916 PMC4311249

[B2] Anand U. Yiangou Y. Sinisi M. Fox M. Macquillan A. Quick T.. (2015). Mechanisms underlying clinical efficacy of Angiotensin II type 2 receptor (AT2R) antagonist EMA401 in neuropathic pain: clinical tissue and *in vitro* studies. Mol. Pain 11:38. 10.1186/s12990-015-0038-x PMC4482278 26111701

[B3] Anderson S. L. Coli R. Daly I. W. Kichula E. A. Rork M. J. Volpi S. A.. (2001). Familial dysautonomia is caused by mutations of the IKAP gene. Am. J. Hum. Genet. 68, 753–758. 10.1086/318808 11179021 PMC1274486

[B4] Ashburn T. T. Thor K. B. (2004). Drug repositioning: identifying and developing new uses for existing drugs. Nat. Rev. Drug Discov. 3, 673–683. 10.1038/nrd1468 15286734

[B5] Ashburner M. Ball C. A. Blake J. A. Botstein D. Butler H. Cherry J. M.. (2000). Gene ontology: tool for the unification of biology. Gene Ontol. Consort. Nat. Genet. 25, 25–29. 10.1038/75556 10802651 PMC3037419

[B6] Azmi A. S. Wang Z. Philip P. A. Mohammad R. M. Sarkar F. H. (2010). Proof of concept: network and systems biology approaches aid in the discovery of potent anticancer drug combinations. Mol. Cancer Ther. 9, 3137–3144. 10.1158/1535-7163.MCT-10-0642 21041384 PMC3058926

[B7] Backes C. Keller A. Kuentzer J. Kneissl B. Comtesse N. Elnakady Y. A.. (2007). Genetrail–advanced gene set enrichment analysis. Nucleic Acids Res. 35, W186–W192. 10.1093/nar/gkm323 17526521 PMC1933132

[B8] Baratloo A. Rouhipour A. Forouzanfar M. M. Safari S. Amiri M. Negida A. (2016). The role of Caffeine in pain management: a brief literature review. Anesth. Pain Med. 6:e33193. 10.5812/aapm.33193 27642573 PMC5018099

[B9] Benjamini Y. Hochberg Y. (1995). Controlling the false discovery rate - a practical and powerful approach to multiple testing. J. R. Stat. Soc. B. 57, 289–300.

[B10] Bonferroni C. E. (1936). Teoria statistica delle classi e calcolo delle probabilita. Pubblicazioni del R Ist. Super. di Sci. Economiche e Commerciali di Firenze 8, 3–62.

[B11] Breivik H. Collett B. Ventafridda V. Cohen R. Gallacher D. (2006). Survey of chronic pain in Europe: prevalence, impact on daily life, and treatment. Eur. J. Pain 10, 287–333. 10.1016/j.ejpain.2005.06.009 16095934

[B12] Bullon P. Alcocer-Gomez E. Carrion A. M. Marin-Aguilar F. Garrido-Maraver J. Roman-Malo L.. (2016). AMPK phosphorylation modulates pain by activation of NLRP3 Inflammasome. Antioxid. Redox Signal. 24, 157–170. 10.1089/ars.2014.6120 26132721 PMC4742979

[B13] Bushati N. Smith J. Briscoe J. Watkins C. (2011). An intuitive graphical visualization technique for the interrogation of transcriptome data. Nucleic Acids Res. 39, 7380–7389. 10.1093/nar/gkr462 21690098 PMC3177207

[B14] Camon E. Magrane M. Barrell D. Binns D. Fleischmann W. Kersey P.. (2003). The gene ontology annotation (GOA) project: implementation of GO in SWISS-PROT, TrEMBL, and InterPro. Genome Res. 13, 662–672. 10.1101/gr.461403 12654719 PMC430163

[B15] Camon E. Magrane M. Barrell D. Lee V. Dimmer E. Maslen J.. (2004). The gene ontology annotation (GOA) database: sharing knowledge in uniprot with gene ontology. Nucleic Acids Res. 32, D262–D266. 10.1093/nar/gkh021 14681408 PMC308756

[B16] Camprubi-Robles M. Mair N. Andratsch M. Benetti C. Beroukas D. Rukwied R.. (2013). Sphingosine-1-phosphate-induced nociceptor excitation and ongoing pain behavior in mice and humans is largely mediated by S1P3 receptor. J. Neurosci. 33, 2582–2592. 10.1523/JNEUROSCI.4479-12.2013 23392686 PMC6619173

[B17] Carbon S. Ireland A. Mungall C. J. Shu S. Marshall B. Lewis S.. (2009). AmiGO: online access to ontology and annotation data. Bioinformatics 25, 288–289. 10.1093/bioinformatics/btn615 19033274 PMC2639003

[B18] Chao H.-T. Davids M. Burke E. Pappas J. G. Rosenfeld J. A. Mccarty A. J.. (2017). A syndromic neurodevelopmental disorder caused by De Novo variants in EBF3. Am. J. Hum. Genet. 100, 128–137. 10.1016/j.ajhg.2016.11.018 28017372 PMC5223093

[B19] Chen Y. C. Auer-Grumbach M. Matsukawa S. Zitzelsberger M. Themistocleous A. C. Strom T. M.. (2015). Transcriptional regulator PRDM12 is essential for human pain perception. Nat. Genet. 47, 803–808. 10.1038/ng.3308 26005867 PMC7212047

[B20] Clifford M. N. Scalbert A. (2000). Ellagitannins – nature, occurrence and dietary burden. J. Sci. Food Agric. 80, 1118–1125. 10.1002/(SICI)1097-0010(20000515)80:7<1118::AID-JSFA570>3.0.CO;2-9

[B21] Cox J. J. Reimann F. Nicholas A. K. Thornton G. Roberts E. Springell K.. (2006). An SCN9A channelopathy causes congenital inability to experience pain. Nature 444, 894–898. 10.1038/nature05413 17167479 PMC7212082

[B22] Cox J. J. Sheynin J. Shorer Z. Reimann F. Nicholas A. K. Zubovic L.. (2010). Congenital insensitivity to pain: novel SCN9A missense and in-frame deletion mutations. Hum. Mutat. 31, E1670–E186. 10.1002/humu.21325 20635406 PMC2966863

[B23] Dawkins J. L. Hulme D. J. Brahmbhatt S. B. Auer-Grumbach M. Nicholson G. A. (2001). Mutations in SPTLC1, encoding serine palmitoyltransferase, long chain base subunit-1, cause hereditary sensory neuropathy type I. Nat. Genet. 27, 309–312. 10.1038/85879 11242114

[B24] Di Cesare Mannelli L. Maresca M. Farina C. Scherz M. W. Ghelardini C. (2015). A model of neuropathic pain induced by sorafenib in the rat: effect of dimiracetam. Neurotoxicology 50, 101–107. 10.1016/j.neuro.2015.08.002 26254739

[B25] Donica C. L. Cui Y. Shi S. Gutstein H. B. (2014). Platelet-derived growth factor receptor-beta antagonism restores morphine analgesic potency against neuropathic pain. PLoS ONE 9:e97105. 10.1371/journal.pone.0097105 24820332 PMC4018247

[B26] Einarsdottir E. Carlsson A. Minde J. Toolanen G. Svensson O. Solders G.. (2004). A mutation in the nerve growth factor beta gene (NGFB) causes loss of pain perception. Hum. Mol. Genet. 13, 799–805. 10.1093/hmg/ddh096 14976160

[B27] Elliott A. M. Smith B. H. Penny K. I. Smith W. C. Chambers W. A. (1999). The epidemiology of chronic pain in the community. Lancet 354, 1248–1252. 10.1016/S0140-6736(99)03057-3 10520633

[B28] Elsaid M. F. Chalhoub N. Kamel H. Ehlayel M. Ibrahim N. Elsaid A.. (2016). Non-truncating LIFR mutation: causal for prominent congenital pain insensitivity phenotype with progressive vertebral destruction? Clin. Genet. 89, 210–216. 10.1111/cge.12657 26285796

[B29] Fisher R. A. (1922). On the interpretation of Chi square from contingency tables, and the calculation of P. J. R. Stat. Soc. 85, 87–94. 10.2307/2340521

[B30] Gastwirth J. L. Glauberman M. (1976). The interpolation of the lorenz curve and gini index from grouped data. Econometrica 44, 479–483. 10.2307/1913977

[B31] Goldberg Y. P. Pimstone S. N. Namdari R. Price N. Cohen C. Sherrington R. P.. (2012). Human mendelian pain disorders: a key to discovery and validation of novel analgesics. Clin. Genet. 82, 367–373. 10.1111/j.1399-0004.2012.01942.x 22845492

[B32] Guelly C. Zhu P. P. Leonardis L. Papic L. Zidar J. Schabhuttl M.. (2011). Targeted high-throughput sequencing identifies mutations in atlastin-1 as a cause of hereditary sensory neuropathy type I. Am. J. Hum. Genet. 88, 99–105. 10.1016/j.ajhg.2010.12.003 21194679 PMC3014370

[B33] Herwig R. Lehrach H. (2006). Expression profiling of drug response-from genes to pathways. Dialogues Clin. Neurosci. 8, 283–293. 17117610 10.31887/DCNS.2006.8.3/rherwig PMC3181826

[B34] Indo Y. Tsuruta M. Hayashida Y. Karim M. A. Ohta K. Kawano T.. (1996). Mutations in the TRKA/NGF receptor gene in patients with congenital insensitivity to pain with anhidrosis. Nat. Genet. 13, 485–488. 10.1038/ng0896-485 8696348

[B35] Intahphuak S. Khonsung P. Panthong A. (2010). Anti-inflammatory, analgesic, and antipyretic activities of virgin coconut oil. Pharm. Biol. 48, 151–157. 10.3109/13880200903062614 20645831

[B36] Janssens K. Goethals S. Atkinson D. Ermanoska B. Fransen E. Jordanova A.. (2014). Human Rab7 mutation mimics features of charcot-marie-tooth neuropathy type 2B in Drosophila. Neurobiol. Dis. 65, 211–219. 10.1016/j.nbd.2014.01.021 24521780

[B37] Julius D. Basbaum A. I. (2001). Molecular mechanisms of nociception. Nature 413, 203–210. 10.1038/35093019 11557989

[B38] Juran J. M. (1975). The non-Pareto principle; Mea culpa. Qual. Prog. 8, 8–9.

[B39] Karadimas C. L. Vu T. H. Holve S. A. Chronopoulou P. Quinzii C. Johnsen S. D.. (2006). Navajo neurohepatopathy is caused by a mutation in the MPV17 gene. Am. J. Hum. Genet. 79, 544–548. 10.1086/506913 16909392 PMC1559552

[B40] Kehlet H. Jensen T. S. Woolf C. J. (2006). Persistent postsurgical pain: risk factors and prevention. Lancet 367, 1618–1625. 10.1016/S0140-6736(06)68700-X 16698416

[B41] Kohonen T. (1982). Self-organized formation of topologically correct feature maps. Biol. Cybern. 43, 59–69. 10.1007/BF00337288

[B42] Kornak U. Mademan I. Schinke M. Voigt M. Krawitz P. Hecht J.. (2014). Sensory neuropathy with bone destruction due to a mutation in the membrane-shaping atlastin GTPase 3. Brain 137, 683–692. 10.1093/brain/awt357 24459106

[B43] Kringel D. Lötsch J. (2015). Pain research funding by the European union seventh framework programme. Eur. J. Pain 19, 595–600. 10.1002/ejp.690 25857368

[B44] Kurth I. Pamminger T. Hennings J. C. Soehendra D. Huebner A. K. Rotthier A.. (2009). Mutations in FAM134B, encoding a newly identified Golgi protein, cause severe sensory and autonomic neuropathy. Nat. Genet. 41, 1179–1181. 10.1038/ng.464 19838196

[B45] Lafreniere R. G. Macdonald M. L. Dube M. P. Macfarlane J. O'driscoll M. Brais B.. (2004). Identification of a novel gene (HSN2) causing hereditary sensory and autonomic neuropathy type II through the study of Canadian genetic isolates. Am. J. Hum. Genet. 74, 1064–1073. 10.1086/420795 15060842 PMC1181970

[B46] Leipold E. Liebmann L. Korenke G. C. Heinrich T. Giesselmann S. Baets J.. (2013). A de novo gain-of-function mutation in SCN11A causes loss of pain perception. Nat. Genet. 45, 1399–1404. 10.1038/ng.2767 24036948

[B47] Libbrecht M. W. Noble W. S. (2015). Machine learning applications in genetics and genomics. Nat. Rev. Genet. 16, 321–332. 10.1038/nrg3920 25948244 PMC5204302

[B48] Liou J. T. Liu F. C. Hsin S. T. Yang C. Y. Lui P. W. (2007). Inhibition of the cyclic adenosine monophosphate pathway attenuates neuropathic pain and reduces phosphorylation of cyclic adenosine monophosphate response element-binding in the spinal cord after partial sciatic nerve ligation in rats. Anesth. Analg. 105, 1830–1837, 10.1213/01.ane.0000287652.42309.5c 18042889

[B49] Lötsch J. Doehring A. Mogil J. S. Arndt T. Geisslinger G. Ultsch A. (2013). Functional genomics of pain in analgesic drug development and therapy. Pharmacol. Ther. 139, 60–70. 10.1016/j.pharmthera.2013.04.004 23567662

[B50] Lötsch J. Ultsch A. (2014). Exploiting the structures of the U-matrix, in Advances in Intelligent Systems and Computing, eds Villmann T. Schleif F. M. Kaden M. Lange M.. (Heidelberg: Springer), 248–257.

[B51] Lötsch J. Ultsch A. (2016a). A machine-learned computational functional genomics-based approach to drug classification. Eur. J. Clin. Pharmacol. 72, 1449–1461. 10.1007/s00228-016-2134-x 27695919

[B52] Lötsch J. Ultsch A. (2016b). Process pharmacology: a pharmacological data science approach to drug development and therapy. CPT Pharmacometrics Syst. Pharmacol. 5, 192–200 10.1002/psp4.12072 27069773 PMC4805871

[B53] Mansour A. R. Farmer M. A. Baliki M. N. Apkarian A. V. (2014). Chronic pain: the role of learning and brain plasticity. Restor. Neurol. Neurosci. 32, 129–139. 10.3233/RNN-139003 23603439 PMC4922795

[B54] Mansouri M. T. Naghizadeh B. Ghorbanzadeh B. (2014). Ellagic acid enhances morphine analgesia and attenuates the development of morphine tolerance and dependence in mice. Eur. J. Pharmacol. 741, 272–280. 10.1016/j.ejphar.2014.08.024 25179576

[B55] Mcgary E. C. Onn A. Mills L. Heimberger A. Eton O. Thomas G. W.. (2004). Imatinib mesylate inhibits platelet-derived growth factor receptor phosphorylation of melanoma cells but does not affect tumorigenicity *in vivo*. J. Invest. Dermatol. 122, 400–405. 10.1046/j.0022-202X.2004.22231.x 15009722

[B56] Miller G. A. (1956). The magical number seven plus or minus two: some limits on our capacity for processing information. Psychol. Rev. 63, 81–97. 10.1037/h0043158 13310704

[B57] Murphy K. P. (2012). Machine Learning: A Probabilistic Perspective. Cambridge, MA: The MIT Press.

[B58] Nahorski M. S. Al-Gazali L. Hertecant J. Owen D. J. Borner G. H. Chen Y. C.. (2015). A novel disorder reveals clathrin heavy chain-22 is essential for human pain and touch development. Brain 138, 2147–2160. 10.1093/brain/awv149 26068709 PMC4511860

[B59] Omura S. Iwai Y. Hirano A. Nakagawa A. Awaya J. Tsuchya H.. (1977). A new alkaloid AM-2282 OF Streptomyces origin. Taxonomy, fermentation, isolation and preliminary characterization. J. Antibiot. 30, 275–282. 10.7164/antibiotics.30.275 863788

[B60] Pareto V. (1909). Manuale Di Economia Politica. Milan: Società Editrice Libraria, Revised and Translated into French as Manuel D'économie Politique. Paris: Giard et Briére.

[B61] Penrod N. M. Cowper-Sal-Lari R. Moore J. H. (2011). Systems genetics for drug target discovery. Trends Pharmacol. Sci. 32, 623–630. 10.1016/j.tips.2011.07.002 21862141 PMC3185183

[B62] Pirim H. Eksioglu B. Perkins A. Yuceer C. (2012). Clustering of high throughput gene expression data. Comput. Oper. Res. 39, 3046–3061. 10.1016/j.cor.2012.03.008 23144527 PMC3491664

[B63] Portenoy R. K. (1992). Cancer pain: pathophysiology and syndromes. Lancet 339, 1026–1031. 10.1016/0140-6736(92)90545-E 1349060

[B64] President's Information Technology Advisory Committee (2005). Report to the President: Computational Science: Ensuring America's Competitiveness. Arlington: National Coordination Office for Information Technology Research and Development.

[B65] R Development Core Team (2008). R: A Language and Environment for Statistical Computing. Vienna.

[B66] Rebhan M. Chalifa-Caspi V. Prilusky J. Lancet D. (1997). Genecards: integrating information about genes, proteins and diseases. Trends Genet. 13:163. 10.1016/S0168-9525(97)01103-7 9097728

[B67] Rice A. S. Dworkin R. H. Mccarthy T. D. Anand P. Bountra C. Mccloud P. I.. (2014). EMA401, an orally administered highly selective angiotensin II type 2 receptor antagonist, as a novel treatment for postherpetic neuralgia: a randomised, double-blind, placebo-controlled phase 2 clinical trial. Lancet 383, 1637–1647. 10.1016/S0140-6736(13)62337-5 24507377

[B68] Riviere J. B. Ramalingam S. Lavastre V. Shekarabi M. Holbert S. Lafontaine J.. (2011). KIF1A, an axonal transporter of synaptic vesicles, is mutated in hereditary sensory and autonomic neuropathy type 2. Am. J. Hum. Genet. 89, 219–230. 10.1016/j.ajhg.2011.06.013 21820098 PMC3155159

[B69] Rotthier A. Baets J. De Vriendt E. Jacobs A. Auer-Grumbach M. Levy N.. (2009). Genes for hereditary sensory and autonomic neuropathies: a genotype-phenotype correlation. Brain 132, 2699–2711. 10.1093/brain/awp198 19651702 PMC2759337

[B70] Salvemini D. Doyle T. Kress M. Nicol G. (2013). Therapeutic targeting of the ceramide-to-sphingosine 1-phosphate pathway in pain. Trends Pharmacol. Sci. 34, 110–118. 10.1016/j.tips.2012.12.001 23318139

[B71] Schaible H. G. (2007). Peripheral and central mechanisms of pain generation. Handb. Exp. Pharmacol. 177, 3–28. 10.1007/978-3-540-33823-9_1 17087118

[B72] Seal R. L. Gordon S. M. Lush M. J. Wright M. W. Bruford E. A. (2011). Genenames.org: the HGNC resources in 2011. Nucleic Acids Res. 39, D514–D519. 10.1093/nar/gkq892 20929869 PMC3013772

[B73] Shannon H. E. Lutz E. A. (2000). Yohimbine produces antinociception in the formalin test in rats: involvement of serotonin(1A) receptors. Psychopharmacology 149, 93–97. 10.1007/s002139900343 10789888

[B74] Sheffer R. Douiev L. Edvardson S. Shaag A. Tamimi K. Soiferman D.. (2016). Postnatal microcephaly and pain insensitivity due to a *de novo* heterozygous DNM1L mutation causing impaired mitochondrial fission and function. Am. J. Med. Genet. A 170, 1603–1607. 10.1002/ajmg.a.37624 26992161

[B75] Slaugenhaupt S. A. Blumenfeld A. Gill S. P. Leyne M. Mull J. Cuajungco M. P.. (2001). Tissue-specific expression of a splicing mutation in the IKBKAP gene causes familial dysautonomia. Am. J. Hum. Genet. 68, 598–605. 10.1086/318810 11179008 PMC1274473

[B76] Sugaya N. Ikeda K. (2009). Assessing the druggability of protein-protein interactions by a supervised machine-learning method. BMC Bioinform. 10:263. 10.1186/1471-2105-10-263 PMC2739204 19703312

[B77] Sugaya N. Ikeda K. Tashiro T. Takeda S. Otomo J. Ishida Y.. (2007). An integrative in silico approach for discovering candidates for drug-targetable protein-protein interactions in interactome data. BMC Pharmacol. 7:10. 10.1186/1471-2210-7-10 17705877 PMC2045083

[B78] Swoboda K. J. Engle E. C. Scheindlin B. Anthony D. C. Jones H. R. (1998). Mutilating hand syndrome in an infant with familial carpal tunnel syndrome. Muscle Nerve 21, 104–111. 9427229 10.1002/(sici)1097-4598(199801)21:1<104::aid-mus13>3.0.co;2-3

[B79] Tasdemir K. Merényi E. (2012). Som-Based Topology Visualization for Interactive Analysis of High-Dimensional Large Datasets. Machine Learning Reports, University of Bielefeld.

[B80] Thulasiraman K. Swamy M. N. S. (1992). Graphs: Theory and Algorithms. New York, NY: Wiley.

[B81] Tsuda M. Kohro Y. Yano T. Tsujikawa T. Kitano J. Tozaki-Saitoh H.. (2011). JAK-STAT3 pathway regulates spinal astrocyte proliferation and neuropathic pain maintenance in rats. Brain 134, 1127–1139. 10.1093/brain/awr025 21371995 PMC4571138

[B82] Tzeng J. Lu H. H. Li W. H. (2008). Multidimensional scaling for large genomic data sets. BMC Bioinform. 9:179. 10.1186/1471-2105-9-179 18394154 PMC2375126

[B83] Ultsch A. (2003). Maps for visualization of high-dimensional data spaces, in Proceeding Workshop on Self-Organizing Maps (WSOM) (Kyushu), 225–230.

[B84] Ultsch A. Kringel D. Kalso E. Mogil J. S. Lötsch J. (2016). A data science approach to candidate gene selection of pain regarded as a process of learning and neural plasticity. Pain 157, 2747–2757. 10.1097/j.pain.0000000000000694 27548044

[B85] Ultsch A. Lötsch J. (2014). Functional abstraction as a method to discover knowledge in gene ontologies. PLoS ONE 9:e90191. 10.1371/journal.pone.0090191 24587272 PMC3935416

[B86] Ultsch A. Lötsch J. (2015). Computed ABC analysis for rational selection of most informative variables in multivariate data. PLoS ONE 10:e0129767. 10.1371/journal.pone.0129767 26061064 PMC4465645

[B87] Ultsch A. Lötsch J. (2017). Machine-learned cluster identification in high-dimensional data. J. Biomed. Inform. 66, 95–104. 10.1016/j.jbi.2016.12.011 28040499 PMC5313598

[B88] Ultsch A. Sieman H. P. (1990). Kohonen's self organizing feature maps for exploratory data analysis, in INNC'90, International Neural Network Conference (Alphen aan den Rijn: Wolters-Kluwer), 305–308.

[B89] van der Maaten L. J. P. Hinton G. E. (2008). Visualizing high-dimensional data using t-SNE. J. Mach. Learn. Res. 9, 2579–2605.

[B90] Verhoeven K. De Jonghe P. Coen K. Verpoorten N. Auer-Grumbach M. Kwon J. M.. (2003). Mutations in the small GTP-ase late endosomal protein RAB7 cause Charcot-Marie-Tooth type 2B neuropathy. Am. J. Hum. Genet. 72, 722–727. 10.1086/367847 12545426 PMC1180247

[B91] Welch S. P. Sim-Selley L. J. Selley D. E. (2012). Sphingosine-1-phosphate receptors as emerging targets for treatment of pain. Biochem. Pharmacol. 84, 1551–1562. 10.1016/j.bcp.2012.08.010 22971335

[B92] Wishart D. S. Knox C. Guo A. C. Cheng D. Shrivastava S. Tzur D.. (2008). DrugBank: a knowledgebase for drugs, drug actions and drug targets. Nucleic Acids Res. 36, D901–D906. 10.1093/nar/gkm958 18048412 PMC2238889

[B93] Wishart D. S. Knox C. Guo A. C. Shrivastava S. Hassanali M. Stothard P.. (2006). DrugBank: a comprehensive resource for in silico drug discovery and exploration. Nucleic Acids Res. 34, D668–D672. 10.1093/nar/gkj067 16381955 PMC1347430

[B94] Woods C. G. Babiker M. O. Horrocks I. Tolmie J. Kurth I. (2015). The phenotype of congenital insensitivity to pain due to the NaV1.9 variant p.L811P. Eur. J. Hum. Genet. 23, 561–563. 10.1038/ejhg.2014.166 25118027 PMC4402639

