# Supplementary figures and images for: Integrated Computational Analysis of Genes Associated with Human Hereditary Insensitivity to Pain. A Drug Repurposing Perspective
Source: Front Mol Neurosci. 2017 Aug 8;10:252. doi: 10.3389/fnmol.2017.00252 (PMC5550731; doi:10.3389/fnmol.2017.00252)

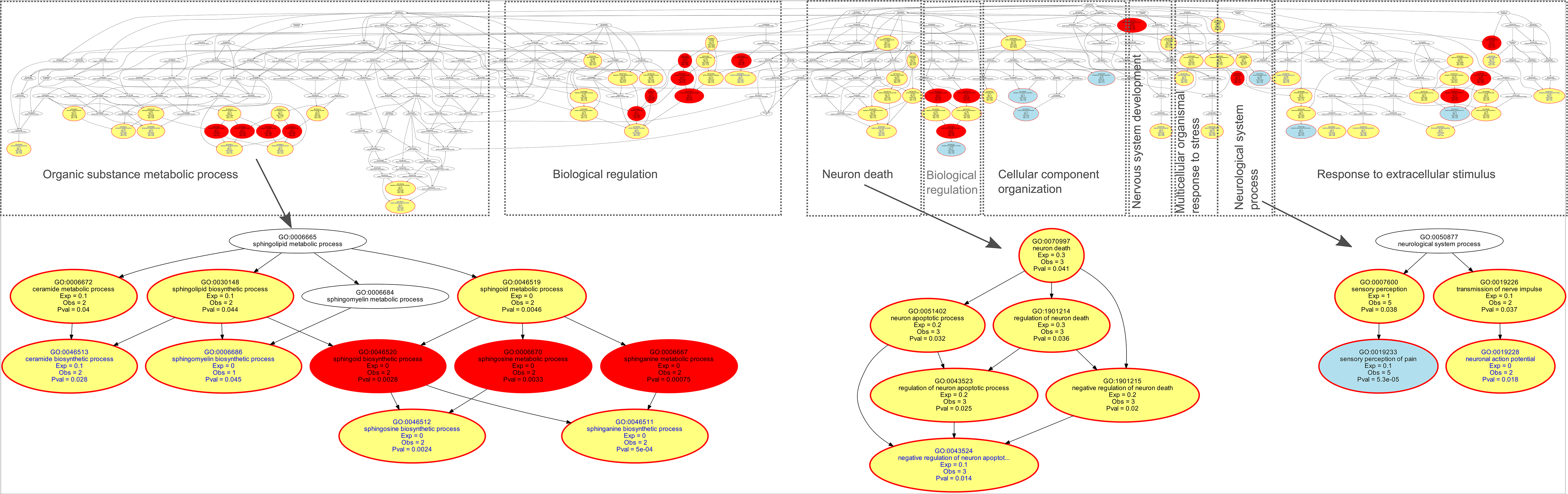

Supplement: Supplementary Figure 1 — This figure shows the top-down representation of the GO terms representing the biological processes in which the set of n = 20 genes causally involved in hereditary absence of pain perception (Table 1) is involved (for an enlarged version, see the Supplementary Figure). The graphical representation follows the standard of the GO knowledgebase, where GO terms are related to each other by “is-a,” “part-of,” and “regulates” relationships forming a polyhierarchy organized in a directed acyclic graph (DAG, Thulasiraman and Swamy, 1992). The figure represents the results of an over-representation analysis with parameters for the p-value threshold, tp = 0.05 and FDR α-correction. (Top) Significant terms are shown as colored circles with the number of member genes, the number of expected genes by chance and the significance of the deviation in the observed from the expected number of genes indicated (yellow = headline, red = significant term located in the polyhierarchy below a functional area). Blue vertices or blue labels, are the most specific terms (leaves of the DAG) at the end of a taxonomy in the polyhierarchy. The biological processes in which these genes are involved can be summarized by seven primary “functional areas” representing the most remarkable nodes with respect to their localization in the polyhierarchy. (Bottom) Zoomed parts of particular functional areas, recreated in a slightly different arrangement to enhance visibility. [file Image1.PNG]
